# Clinical significance of visual cardiac ^18^F-FDG uptake in advanced non-small cell lung cancer

**DOI:** 10.1186/s40644-024-00800-w

**Published:** 2024-11-18

**Authors:** Kosuke Hashimoto, Kyoichi Kaira, Hisao Imai, Ou Yamaguchi, Atsuto Mouri, Ayako Shiono, Yu Miura, Kunihiko Kobayashi, Hiroshi Kagamu, Ichiei Kuji

**Affiliations:** 1 Department of Respiratory Medicine, International Medical Center, Saitama Medical University, 1397-1 Yamane, Hidaka city/Saitama, 350-1298 Japan; 2 Department of Nuclear Medicine, International Medical Center, Saitama Medical University, 1397-1 Yamane, Hidaka city/Saitama, 350-1298 Japan

## Abstract

**Background:**

Two-deoxy-2-[fluorine-18]-fluoro-d-glucose (^18^F-FDG) positron emission tomography (PET) is useful for detecting malignant lesions; however, the clinical significance of cardiac ^18^F-FDG uptake in patients with cancer remains unclear. This preliminary study explored the relationship between cardiac ^18^F-FDG uptake and advanced diseases such as cancer cachexia in non-small cell lung cancer (NSCLC).

**Methods:**

Forty-three patients with advanced NSCLC who underwent ^18^F-FDG PET and complained of weight loss before the first-line systemic therapy were retrospectively included in this study. Visual assessment using a 5-point scale based on ^18^F-FDG uptake was performed; a cut-off score of 3 was determined, a low score was 1, 2, or 3, and a high score was 4 or 5).

**Results:**

High and low visual cardiac ^18^F-FDG uptakes were observed in 27 (62.8%) and 16 (37.2%) patients, respectively. Of the 43 patients, 17 (39.5%) definitely had cachexia, and 26 (60.5%) did not. A low visual score and standardized uptake value_max_ for cardiac ^18^F-FDG uptake were significantly associated with high metabolic tumor activity (*p* = 0.009, and* p* = 0.009, respectively) and a high neutrophil-to-lymphocyte ratio (*p* = 0.016, and *p* = 0.047, respectively), whereas a low visual score for cardiac ^18^F-FDG uptake and high metabolic tumor activity were significantly associated with cachexia (*p* = 0.004). The amount of cardiac ^18^F-FDG accumulation depicted a close relationship with body mass index, low weight loss, and inflammation. The combination of cachexia and low visual cardiac ^18^F-FDG uptake was identified as a significant predictor for poor overall survival (OS) (*p* = 0.034).

**Conclusion:**

Decreased visual cardiac ^18^F-FDG uptake was associated with poor nutritional status and OS, and cachexia in patients with advanced NSCLC.

## Introduction

Two-deoxy-2-[fluorine-18]-fluoro-d-glucose (^18^F-FDG) positron emission tomography (PET) is commonly used for diagnosis, staging, and therapeutic monitoring of patients with cancer. ^18^F-FDG accumulates in organs that require high glucose utilization, such as the heart, brain, and liver. The mechanism of ^18^F-FDG uptake within tumor cells via glucose transporter 1 is well known, and ^18^F-FDG accumulation is quantified using the standardized uptake value (SUV) [1]. Although SUV is helpful for the assessment of tumor glycolytic capacity, increased glucose consumption within tumor tissues can disturb systemic metabolic flux and may affect ^18^F-FDG uptake in different organs.

Cancer cachexia is a multifactor syndrome characterized by weight loss, skeletal muscle wasting, and adipose tissue atrophy [2]. Cachexia is frequently observed in patients with advanced-stage cancer and may lead to worse outcomes, disease aggravation, and increased mortality. A recent review described how imaging modalities for quantitative and qualitative change in adipose tissue, organs, and muscle compartments are assessed for the diagnosis and monitoring of cancer cachexia [3]. Olaechea et al. [4] reported a significant positive relationship between cancer-associated weight loss at diagnosis and increased primary tumor SUV_max_ for ^18^F-FDG uptake in patients with non-small cell lung cancer (NSCLC). An experimental study has shown that ^18^F-FDG uptake is higher in cachexia-inducing tumor cells than in non-cachexia tumor cells [5]. Potentially, escalated tumor glucose consumption, such as high ^18^F-FDG uptake, is associated with the presence of a tumor phenotype that induces weight loss [4]. Previous studies have reported that body weight is closely related to physiological ^18^F-FDG uptake by the liver [6, 7]. Nakamoto et al. [8] reported that patients with cancer with decreased ^18^F-FDG uptake in the liver appear to have cancer cachexia and poor outcomes. Although ^18^F-FDG normally accumulates in the liver, brain, and heart, there are limited data regarding the clinical significance of ^18^F-FDG uptake in cardiac metabolism in patients with cancer. A recent study found an increased cardiac ^18^F-FDG uptake in patients with Hodgkin’s lymphoma [9]. However, the relationship between cardiac ^18^F-FDG uptake and general conditions such as cancer cachexia remains unclear. To date data is lacking on how cardiac glucose metabolism is affected by inflammatory changes, nutrition, the immunological environment, and the general condition of patients with cancer. Although systemic therapeutic agents are administered to patients with advanced cancer, they can affect glucose metabolism in different organs, such as the liver and heart.

In this study, we examined whether cardiac ^18^F-FDG uptake was potentially affected by different factors, with a focus on cachexia, in patients with advanced lung cancer who received systemic treatment. In addition, we explored the clinical significance of cardiac ^18^F-FDG uptake in patients with advanced NSCLC and compared it with cachexia and other variables.

## Methods

### Patients

Between January 2021 and June 2021, 58 consecutive patients with advanced NSCLC who received systemic treatment at our institution and complained of weight loss before first-line treatment were retrospectively registered in a pilot study for cardiac ^18^F-FDG uptake. Fifteen of these 58 patients were excluded from this study because they lacked a PET information, had inappropriate timing of PET, and histology of combined small cell carcinoma. Thus, 43 patients were eligible for our study. The inclusion criteria were as follows: 1) histologically diagnosed NSCLC; 2) advanced stage IV disease; 3) receiving any systemic treatment, including chemotherapy, immunotherapy, or molecular targeting therapy; 4) receiving ^18^F-FDG PET immediately before initial treatment; 5) complaint of weight loss within the previous 3 months; and availability of detailed body weight records. Clinical data were extracted from medical records.

This study was approved by the Institutional Ethics Committee of the International Medical Center of Saitama Medical University. The requirement for written informed consent was waived by the Ethics Committee of Saitama Medical University due to the retrospective nature of the study [10].

### Treatment and evaluation

All patients were treated with a combination of nivolumab, a programmed death-1 (PD-1) blockade, and ipilimumab, an anti-cytotoxic T-lymphocyte-associated antigen 4 antibody, combined chemotherapy with PD-1 blockade, epidermal growth factor receptor-tyrosine kinase inhibitors (EGFR-TKIs), or platinum-based chemotherapy. Physical examination, complete blood count, biochemical testing, and adverse event assessment were performed by a chief physician. Toxicity was graded based on the Common Terminology Criteria for Adverse Events, version 4.0. The tumor response was examined using the Response Evaluation Criteria in Solid Tumors version [11].

### PET imaging and data analysis

Patients fasted for at least 6 h before ^18^F-FDG PET, which was performed using a PET/computed tomography (CT) scanner. Three-dimensional data acquisition was initiated 60 min after FDG injection. Eight bed positions were selected based on the imaging range. Attenuation-corrected transverse images obtained with ^18^F-FDG were reconstructed using an ordered-subset expectation–maximization algorithm based on the point-spread function into 168 × 168 matrices with a slice thickness of 2.00 mm.

For the semi-quantitative analysis, the SUV was examined based on the ^18^F-FDG injected dose, patient weight, and cross-calibration factor between the PET and the dose calibrator. SUV was defined as: SUV = radioactive concentration in the volume of interest (VOI) (MBq/g)/injected dose (MBq)/patient’s weight(g). CT for initial staging was performed using an intravenous contrast medium and board-certified radiologists interpreted the images. We used Syngo. via (SIEMENS Healthineers Co. Ltd., Japan) on a Windows workstation to semi-automatically calculate the maximum SUV (SUV_max_), metabolic tumor volume (MTV), and total lesion glycolysis (TLG), defined as MTV multiplied by the SUV_mean_, of each lesion using SUV thresholds obtained by the SUV in the liver VOI. Each threshold was defined as the average of 1.5 × SUV (SUV_mean_) plus 2 × SD of SUV in the liver. These SUV thresholds were the optimum values for generating a 3D VOI in which the entire tumor mass was completely enclosed in all cases using the CT image as the reference. Regions of activity other than the tumors, including the myocardium, gastrointestinal tract, kidneys, and urinary tract, were manually eliminated according to the orientation provided by a board-certified nuclear medicine physician.

In this study, a five-point scale (5-PS) based on ^18^F-FDG uptake was used for therapeutic monitoring of patients with malignant lymphoma and NSCLC [12, 13]. The 5-PS score was defined as follows:

1. No uptake
2. Uptake ≦ mediastinum
3. Uptake > mediastinum and ≦ liver
4. Uptake moderately higher than the liver
5. Uptake markedly higher than the liver and/or new lesions.

The optimal cut-off values for the SUV_max_, MTV, and TLG were the median values, and those markers with a value greater than the cut-off value were determined to have a high expression.

### Assessment of the inflammatory and nutritional indices

Clinical and biological data (total protein, albumin, and C-reactive protein [CRP] levels; white blood cell, neutrophil, platelet, and lymphocyte counts; and height and weight) were extracted from the medical records and analyzed. Six indices reflecting systemic inflammatory and nutritional status based on a previous study [14] were calculated at baseline within 1 week of the first cycle of each treatment. The inflammatory indices were: 1) NLR = neutrophil count/lymphocyte count and 2) PLR = platelet count/lymphocyte count. The nutritional indices were as follows: 1) prognostic nutritional index (PNI) = 10 × albumin (g/dL) + 0.005 × lymphocyte count and 2) Glasgow prognostic score (GPS). The GPS was tabulated as: 0 = no abnormal values (good), 1 = one abnormal value (intermediate); and 2 = two abnormal values (poor). Abnormal values included C-reactive protein (CRP) > 10 mg/mL and albumin < 3.5 g/dL. A GPS of 0 was defined as low, and a GPS of 1 or 2 was defined as high. The optimal cutoff values for NLR, PLR, and PNI were the median values, and thoseindices with values greater than the cutoff value were defined as high.

### Statistical analysis

Statistical significance was set at *p* < 0.05. Fisher’s exact test was used to examine the association between two categorical variables. Correlations among SUV_max_, MTV, TLG, and ^18^F-FDG uptake were analyzed using Pearson’s rank tests. Progression-free survival (PFS) was defined as the time from initial treatment to disease progression or death. Overall survival (OS) was defined as the time from initial treatment to death from any cause. The Kaplan–Meier method was used to estimate survival as a function of time, and survival differences were analyzed using the log-rank test. The univariate and multivariate analyses of different variables were performed using logistic regression and the COX hazard model. The step wise method was used in multivariate analysis. All statistical analyses were performed using GraphPad Prism (v.7.0e; GraphPad Software, San Diego, CA, USA) and JMP Pro 16.0 (SAS Institute Inc., Cary, NC, USA).

## Results

### Patient demographics

**Fig. 1:**
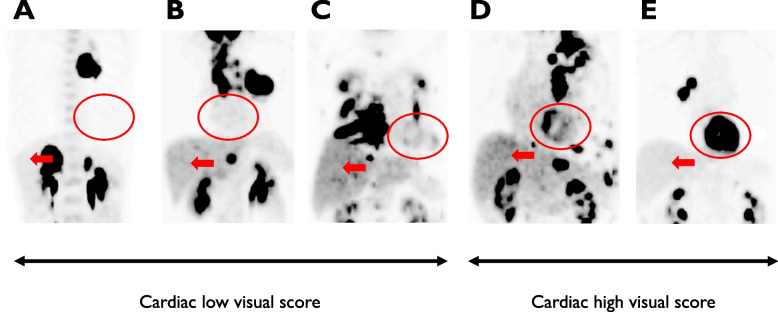
^18^F-FDG PET findings before first-line therapeutic agent administration in patients with advanced NSCLC. PET imaging before any treatment showing representative images based on the definitions of the five-point scale (5-PS) scores using cardiac ^18^F-FDG uptake (red dotted frame, cardiac lesion; red arrow, liver). Score of 1: no uptake (**A**); score of 2: uptake lower than the mediastinum (**B**); score of 3: uptake higher than the mediastinum and lower than the liver (**C**); score of 4: uptake moderately higher than the liver (**D**); and score of 5: uptake markedly higher than the liver (**E**). Low cardiac visual scores are 1, 2, or 3, and high cardiac visual scores are 4 or 5. Abbreviations: ^18^F-FDG PET, two-deoxy-2-[fluorine-18]-fluoro-d-glucose positron emission tomography^1^; NSCLC, non-small cell lung cancer

A total of 43 patients were registered in this study: 17 (39.5%) had cachexia, whereas cachexia was not observed in 26 (60.5%) patients. Cardiac ^18^F-FDG uptake was visually assessed based on the 5-PS scores [12, 13]. A previous study revealed that the optimal score for ^18^F-FDG uptake cut-offs for outcome in patients with NSCLC was 3 [13]. Therefore, a score of 3 was chosen as the cutoff point for further analysis, and all patients were divided into two groups; patients with scores of 1,2 or 3 were in the low group and those with scores of 4 or 5 were in the high group [13] (Fig. 1). Twenty-seven (62.8%) patients had high visual cardiac ^18^F-FDG uptake and 16 (37.2%) patients had low accumulation. The patient demographics according to visual cardiac ^18^F-FDG uptake are listed in Table 1. The median value of SUV_max_ by tumor and cardiac ^18^F-FDG uptake in 41 patients with stage IV was 9.5 (range, 3.2–21.7) and 4.3 (range, 1.6–11.8), respectively, indicating that the SUV_max_ of tumor ^18^F-FDG uptake was significantly higher than that of cardiac ^18^F-FDG uptake (*p* < 0.001). The median values of NLR, PLR, and PNI were 3.8 (range, 1.8–6.5), 213.5 (range, 117.4–295.1), and 43.9 (range, 40.7–63.0).

**Table 1:** Patient’s demographics according to visual cardiac ^18^F-FDG uptake

Different variables		Total	Visual cardiac ^18^F-FDG accumulation		
		*n* = 43	High (*n* = 27)	Low (*n* = 16)	*p*-value
**Age**	< 70 yr / ≥ 70 yr	17 / 26	9 / 18	8 / 8	0.280
**Gender**	Male / Female	29 / 14	18 / 9	11 / 5	0.888
**ECOG PS**	2–3	38 / 4	26 / 1	12 / 3	0.085
**Smoking**	Yes / No	34 / 9	19 / 8	15 / 1	0.069
**Histology**	AC / Non-AC	30 / 13	22 / 5	8 / 8	**0.030**
**Stage**	Non-IV / IV	2 / 41	1 / 26	1 / 15	0.702
**Cachexia**	Yes / No	17 / 26	6 / 21	11 / 5	**0.004**
**NLR**	High / Low	22 / 21	10 / 17	12 / 4	**0.016**
**PLR**	High / Low	22 / 21	12 / 15	10 / 6	0.252
**PNI**	High / Low	22 / 21	15 / 12	7 / 9	0.454
**GPS**	0 / 1–2	25 / 18	18 / 9	7 / 9	0.141
**PD-L1**	≧50% / 1–49% / < 1% / Unknown	11 / 16 / 15 / 1	7 / 11 / 9 / 0	4 / 5 / 6 / 1	0.575
**First-line**	ICI / TKI / Chemo	30 / 10 / 3	20 / 6 / 1	10 / 4 / 2	0.511
**BMI**	High / Low	27 / 16	19 / 8	8 / 8	0.182
**LDH**	High / Low	17 / 26	9 / 18	8 / 8	0.280
**CRP**	High / Low	21 / 21	12 / 15	9 / 6	0.334
**BUN**	High / Low	22 / 21	15 / 12	7 / 9	0.454
**PLT**	High / Low	22 / 21	11 / 16	11 / 5	0.076
**Tumor SUV**_**max**_	High / Low	21 / 21	11 / 15	10 / 6	0.204
**Tumor MTV**	High / Low	19 / 19	7 / 15	12 / 4	**0.009**
**Tumor TLG**	High / Low	19 / 19	7 / 15	12 / 4	**0.009**

*Abbreviations*: *SUV*_*max*_ the maximum of standardized uptake value, *MTV* Metabolic tumor volume, *TLG* Total lesion glycolysis, *ECOG PS* Eastern cooperative oncology group performance status, *AC* Adenocarcinoma, *non-AC* non-adenocarcinoma, *CRP* C-reactive protein, *PD-L1* Programmed death ligand-1, *NLR* Neutrophil to lymphocyte ratio, *PLR* Platelet to lymphocyte ratio, *SII* Systemic immune inflammation index, *PNI* Prognostic nutrition index, *ALI* Advanced lung cancer inflammation index, *GPS* Glasgow prognostic score, *ICI* Immune checkpoint inhibitor, *TKI* Tyrosine kinase inhibitor, *Chemo* Chemotherapy, *BMI* Body mass index, *LDH* Lactate dehydrogenase, *BUN* blood urea nitrogen

Low visual cardiac ^18^F-FDG uptake was significantly observed in patients with non-AC (adenocarcinoma) (*p* = 0.030), cachexia (*p* = 0.004), high NLR (*p* = 0.016), high tumor MTV (*p* = 0.009), and high tumor TLG (*p* = 0.009). Table 2 presents the patient demographics according to cardiac ^18^F-FDG uptake by SUV_max_. Patients with a low SUV_max_ for cardiac ^18^F-FDG exhibited a significantly higher NLR (*p* = 0.047), MTV (*p* = 0.009), and TLG (*p* = 0.009) than those with a high SUV_max_. Regarding genetic alterations, *EGFR* mutation and anaplastic lymphoma kinase rearrangement were positive in nine and one patients, respectively. For first-line treatment, the nine patients harboring *EGFR* mutations received EGFR-TKIs (three patients in gefitinib and six patients in osimertinib), and one patient with anaplastic lymphoma kinase rearrangement was treated with alectinib. Thirty patients were treated with immune checkpoint inhibitors (ICIs), including 21 patients who received ipilimumab plus nivolumab, 5 patients received pembrolizumab, and 4 patients who received carboplatin, paclitaxel, bevacizumab and atezolizumab. Two patients received carboplatin plus nab-paclitaxel and one patient received cisplatin plus pemetrexed. The metastatic status of 41 patients with stage IV revealed that there are 10 patients with pulmonary metastasis, 12 patients with pleural metastasis, 6 patients with brain metastasis, 17 patients with bone metastasis, 5 patients with liver metastasis, 5 patients with adrenal metastasis, 12 patients with lymph node metastasis, 2 patients with skin metastasis, and one patient with other.

**Table 2:** Patient’s demographics according to cardiac ^18^F-FDG uptake

Different variables		Total	cardiac ^18^F-FDG by SUV_max_		
		*n* = 43	High (*n* = 22)	Low (*n* = 21)	*p*-value
**Age**	< 70 yr / ≥ 70 yr	17 / 26	8 / 14	9 / 12	0.663
**Gender**	Male / Female	29 / 14	14 / 8	15 / 6	0.586
**ECOG PS**	0–1 / 2–3	38 / 4	21/1	17 / 3	0.249
**Smoking**	Yes / No	34 / 9	15 / 7	19 / 2	0.072
**Histology**	AC / Non-AC	30 / 13	18 / 4	12 / 9	0.078
**Stage**	Non-IV / IV	2 / 41	1 / 21	1 / 20	0.973
**NLR**	High / Low	22 / 21	8 / 14	14 / 7	**0.047**
**PLR**	High / Low	22 / 21	10 / 12	12 / 9	0.443
**PNI**	High / Low	22 / 21	13 / 9	9 / 12	0.287
**GPS**	0 / 1–2	25 / 18	15 / 7	10 / 11	0.172
**BMI**	High / Low	27 / 16	15 / 7	12 / 9	0.454
**Cachexia**	Yes / No	17 / 26	6 / 16	11 / 10	0.092
**Weight loss**	Yes / No	15 / 21	5 / 13	10 / 8	0.091
**Tumor SUV**_**max**_	High / Low	21 / 21	10 / 11	11 / 10	0.758
**Tumor MTV**	High / Low	19 / 19	6 / 14	13 / 5	**0.009**
**Tumor TLG**	High / Low	19 / 19	6 / 14	13 / 5	**0.009**

*Abbreviations*: *SUV*_*max*_, the maximum of standardized uptake value, *MTV* Metabolic tumor volume, *TLG* Total lesion glycolysis, *ECOG PS* Eastern cooperative oncology group performance status, *AC* Adenocarcinoma; non-AC, non-adenocarcinoma, *CRP* C-reactive protein, *PD-L1* Programmed death ligand-1, *NLR* Neutrophil to lymphocyte ratio, *PLR* Platelet to lymphocyte ratio, *SII* Systemic immune inflammation index, *PNI* Prognostic nutrition index, *ALI* Advanced lung cancer inflammation index, *GPS* Glasgow prognostic score, *BMI* Body mass index

The findings of electrocardiogram showed that there are 8 patients with complete right bundle block, one patient with Movitz type II atrioventricular block, one patient with type 1 atrioventricular block, 4 patients with left ventricular hypertrophy, 2 patients with old myocardial infarction, one patient with left bundle block, one patient with supraventricular premature contractions, 2 patients with atrial fibrillation, one patient with premature ventricular contractions, and one patient with pacemaker. Regarding the information of diabetes mellitus, 7 patients with diabetes mellitus receive any medicine, and hemoglobin A1c of more than 6.5% was observed in 8 patients. The median value of blood sugar level before the performance of ^18^F-FDG PET imaging was 111.5 mg/dl, ranging from 88 to 179 mg/dl.

### Relationship between ^18^F-FDG uptake and cachexia

**Fig. 2:**
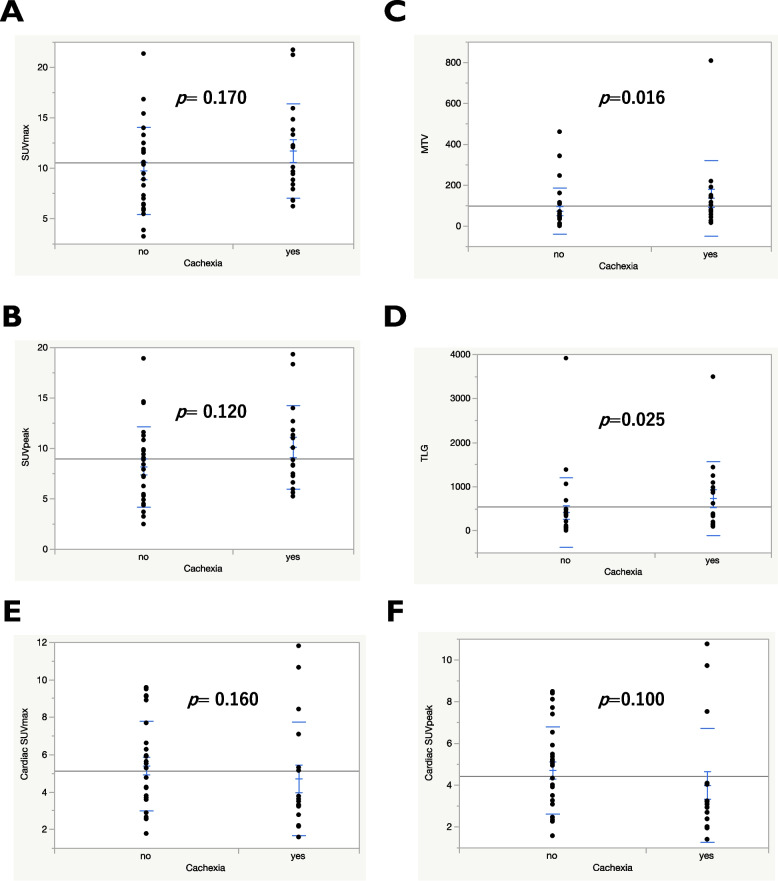
Comparison of SUV_max_ (**A**), SUV_peak_ (**B**), MTV (**C**), TLG (**D**), cardiac SUV_max_ (**E**), and cardiac SUV_peak_ (**F**) according to cachexia presence. MTV and TLG were significantly higher in patients with cachexia than those without cachexia. Abbreviations: SUV, standardized uptake value; MTV, metabolic tumor volume; TLG, total lesion glycolysis

The quantitative values of ^18^F-FDG uptake on PET were compared based on the presence or absence of cachexia (Fig. 2). No statistically significant differences in SUV_max_ (Fig. 2A) or SUV_peak_ (Fig. 2B) for ^18^F-FDG uptake were observed between patients with and without cachexia. However, the MTV (Fig. 2C) and TLG (Fig. 2D) for ^18^F-FDG uptake were significantly higher in patients with cachexia than in those without cachexia.The cardiac SUV_max_ (Fig. 2E) and SUV_peak_ (Fig. 2F) did not differ according to the presence of cachexia.

### Correlation of ^18^F-FDG uptake with BMI or weight loss

**Fig. 3:**
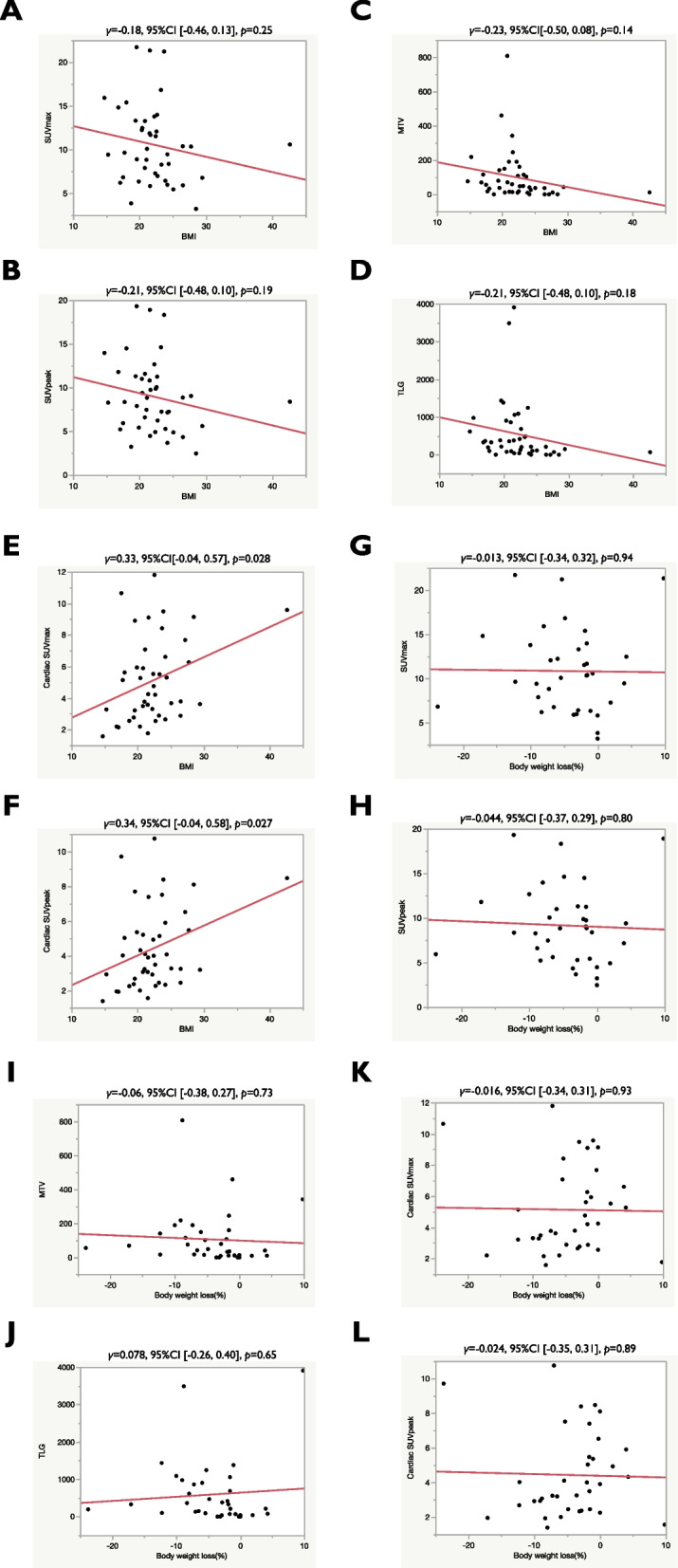
Correlation of ^18^F-FDG uptake with different variables. Pearson’s correlations of BMI with SUV_max_ (**A**), SUV_peak_ (**B**), MTV (**C**), TLG (**D**), cardiac SUV_max_ (**E**), and cardiac SUV_peak_ (**F**) were performed. SUV_max_ (**G**), SUV_peak_ (**H**), MTV (**I**), TLG (**J**), cardiac SUV_max_ (**K**), and cardiac SUV_peak_ (**L**) correlated with weight loss (%).Abbreviations: ^18^F-FDG, Two-deoxy-2-[fluorine-18]-fluoro-d-glucose; BMI, body mass index; SUV, standardized uptake value; MTV, metabolic tumor volume; TLG, total lesion glycolysis; 95% CI, 95% confidence interval

Figure 3 presents the correlation among ^18^F-FDG uptake, BMI, and body weight loss. The amount of ^18^F-FDG accumulation based on SUV_max_, SUV_peak_, MTV, and TLG was not significantly correlated with BMI or weight loss. Although there was no close correlation between weight loss and cardiac SUV_max_ or SUV_peak_, the amount of ^18^F-FDG uptake according to the cardiac SUV_max_ was significantly correlated with BMI (Fig. 3E).

### Relationship between visual cardiac ^18^F-FDG uptake and different variables

**Fig. 4:**
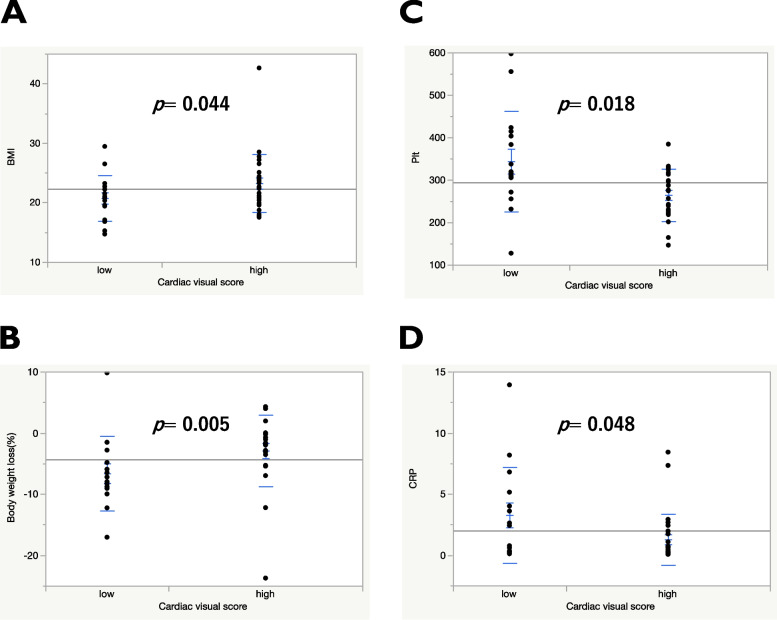
Comparison of BMI (**A**), weight loss (%) (**B**), Plt (**C**), and CRP (**D**) according to cardiac visual score on ^18^F-FDG uptake. Abbreviations: ^18^F-FDG, Two-deoxy-2-[fluorine-18]-fluoro-d-glucose; BMI, body mass index; Plt, platelet; CRP, C-reactive protein

This study examined the close association between visual cardiac ^18^F-FDG uptake and different variables such as white blood cell count, neutrophil, lymphocyte, platelet, total protein, albumin, lactate dehydrogenase, blood urea nitrogen, creatinine, CRP, GPS, NLR, PLR, and PNI. High BMI (Fig. 4A), low weight loss (Fig. 4B), low platelet count (Fig. 4C), and low CRP levels (Fig. 4D) were significantly associated with high visual cardiac ^18^F-FDG uptake. Besides, the cardiac SUV_max_ was not significantly correlated with tumor SUV [r = -0.144, 95% confidence interval (CI) -0.429 to 0.166, *p* = 0.361], MTV (r = -0.219, 95% CI -0.504 to 0.107, *p* = 0.181), and TLG (r = -0.290, 95% CI -0.558 to 0.032, *p* = 0.077). There was not significant correlation between cardiac SUV_max_ and NLR (r = -0.005, 95% CI -0.301 to 0.299, *p* = 0.997).

### Survival analysis according to cardiac visual score and cachexia

**Fig. 5:**
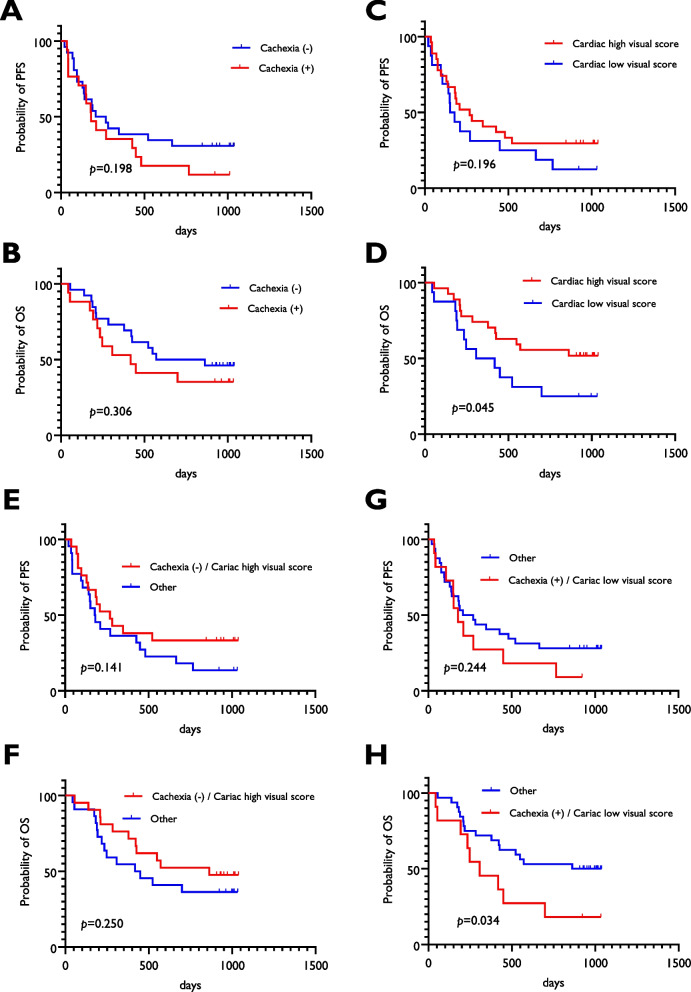
Kaplan–Meier curves of PFS and OS according to cachexia and cardiac visual scores. No statistically significant difference for the PFS (**A**) and OS (**B**) was observed between the patients with and without cachexia. There was no significant difference for PFS between a cardiac high and low visual score (**C**); however, the patients with a low visual score yielded a worse OS than those with a high score (**D**). The absence of cachexia and a cardiac high visual score were not identified as a prognostic predictor of PFS (**E**) and OS (**F**), whereas, cachexia and a cardiac low visual score depicted a significant predictor for OS (**H**) but not PFS (**G**). Abbreviations: PFS, progression-free survival; OS overall survival

The follow-up period was 549 (range, 43–1049) days. The median PFS and OS after the initial treatment were 208 and 549 days, respectively. Thirty-three patients experienced disease progression, and 25 died due to the primary disease. The Kaplan–Meier survival curve according to the cardiac visual score and cachexia is presented in Fig. 5. No statistically significant differences in PFS [median survival time (MST), 180 days vs. 238 days] (Fig. 5A) or OS (MST, 418 days vs. 716 days) (Fig. 5B) were observed between patients with and without cachexia. Although there was no significant difference in PFS (MST, 269 days vs. 164 days) (Fig. 5C) between patients with high and low cardiac visual scores, a significant difference in OS (MST, not reached vs. 362 days) (Fig. 5D) was observed between the two groups.

The absences of cachexia with a high cardiac visual score was not identified as a prognostic predictor for PFS (MST, 269 days vs. 179 days) (Fig. 5E) and OS (MST, 862 days vs. 733 days) (Fig. 5F), whereas cachexia and a low cardiac visual score was a significant predictor for OS (MST, 307 days vs. 950 days) (Fig. 5H) but not PFS (MST, 178 days vs. 238 days) (Fig. 5G).

Next, univariate analysis of all patients identified histology, NLR, GPS, cardiac visual score, cardiac SUV_max_, and, tumor TLG, as significant predictors of OS (Table 3). The univariate log-rank test enabled screening for variables with p < 0.05 for subsequent multivariate analysis. As a significant predictor for PFS was not observed, we examined the multivariate analysis for predictors of OS. Multivariate analysis identified NLR and cardiac SUV_max_ as independent predictors of OS. Moreover, the cut off values for cardiac SUV_max_ and NLR were examined by receiver operating characteristic curve (ROC) analysis and sensitivity and specificity were calculated to determine the optimal cut-off value for differentiating cachexia ( +) from cachexia (-) using ROC curves. As a result, the optimal cutoff values for the cardiac SUV_max_ and NLR as determined by ROC curves analyses were 4.2 (sensitivity: 58.8%, specificity: 57.7%) and 3.9 (sensitivity: 64.7%, specificity: 69.3%), and the areas under the curve in the ROC analysis were 0.563 (cardiac SUV_max_) and 0.660 (NLR). These cut-off values were almost similar to those of median cut-off value. Therefore, the results of survival analyses for the cardiac SUV_max_ and NLR were corresponding to those for Table 3.

**Table 3:** Univariate and multivariate survival analysis

Different variables		PFS		OS			
		Univariate		Univariate		Multivariate	
		MST (days)	*p*-value	MST (days)	*p*-value	HR (95%CI)	*p*-value
**Age**	< 70y ≧70y	189 209	0.701	NR 474.5	0.288		
**Gender**	Male Female	189 240	0.529	449 NR	0.074		
**PS**	0–1 2–3	209 315	0.780	560 342	0.513		
**Histology**	AC Others	276 137	0.174	NR 234	**0.023**	1.372 (0.524–3.591)	0.523
**NLR**	High Low	150 210	0.545	296 NR	**0.007**	2.745 (1.049–7.179)	**0.032**
**PLR**	High Low	165 282	0.844	559 549	0.955		
**PNI**	High Low	240 178	0.701	560 449	0.494		
**GPS**	0 1–2	270 145	0.107	NR 306	**0.008**	2.392 (0.916–6.245)	0.075
**BMI**	High Low	208 225	0.432	523 NR	0.414		
**Cachexia**	Yes No	180 239	0.198	418 717	0.306		
**Cardiac** **FDG (visual)**	High Low	268 165	0.196	NR 363	**0.045**	0.144 (0.024–0.855)	0.060
**Cardiac SUV**_**max**_	High Low	308 178	0.101	NR 378	**0.015**	0.088 (0.013–0.584)	**0.028**
**Tumor SUV**_**max**_	High Low	178 210	0.753	699 49	0.677		
**Tumor MTV**	High low	151 347	0.433	307 NR	0.112		
**Tumor TLG**	High Low	149 427	0.196	247 NR	**0.019**	1.529 (0.563–4.149)	0.401
**First-line regimen**	ICI Non-ICI	182 270	0.742	781 418	0.350		

*Abbreviations*: *MST* median survival time (days), *HR* hazard ratio, *95% CI* 95% confidence interval, *NR* not reached, *SUV*_*max*_ the maximum of standardized uptake value, *MTV* metabolic tumor volume, *TLG* total lesion glycolysis, *ECOG PS* eastern cooperative oncology group performance status, *AC* adenocarcinoma, *non-AC* non-adenocarcinoma, *CRP* C-reactive protein, *PD-L1* programmed death ligand-1, *NLR* neutrophil to lymphocyte ratio, *PLR* platelet to lymphocyte ratio, *SII* systemic immune inflammation index, *PNI* prognostic nutrition index, *ALI* advanced lung cancer inflammation index, *GPS* Glasgow prognostic score, *BMI* body mass index, *ICI* immune checkpoint inhibitor, *PFS* progression-free survival, *OS* overall survival, bold type, statistically significance

## Discussion

To our knowledge, this was the first pilot study to evaluate the relationship between clinical markers and cardiac ^18^F-FDG uptake in patients with advanced NSCLC. In our approach, both the visual score and SUV_max_ were used to measure cardiac ^18^F-FDG accumulation. Measuring the visual score is easy and convenient for physicians, as reported previously reported [13]. A low level of visual cardiac ^18^F-FDG uptake was closely associated with cachexia, high NLR, and high metabolic tumor volume (MTV and TLG). Although there was a similar trend between the visual score and SUV_max_ for cardiac ^18^F-FDG uptake, visual cardiac ^18^F-FDG uptake seemed more relevant than SUV_max_ for cardiac ^18^F-FDG uptake. In particular, a low level of visual cardiac ^18^F-FDG uptake was significantly correlated with low BMI, high weight loss, and increased inflammation, suggestive of cancer cachexia. We found that decreased cardiac ^18^F-FDG uptake was closely related to negative prognostic factors such as high NLR, MTV, and TLG in advanced NSCLC [14, 15], and that patients with cancer cachexia exhibited a higher metabolic tumor volume by MTV or TLG.

As this study included heterogeneous first-line regimens, our survival analysis identified a limitation of therapeutic bias. The survival analysis with the limited sample size showed that low visual cardiac ^18^F-FDG uptake was a predictor of poor OS, particularly for patients with cachexia. Considering the exploratory results of our study, a low visual score for cardiac ^18^F-FDG uptake potentially indicated a close relationship with cancer cachexia, low nutrition, and increased metabolic tumor activity. Further investigation using a larger sample size is warranted to confirm the results of our preliminary study.

An experimental study using lung carcinoma cells indicated that the disruption of muscle oxidative metabolism is associated with cancer cachexia progression in mice [16]. To measure the oxidative stress on myocardial metabolism, myocardial-SUV on ^18^F-FDG uptake was investigated and was closely correlated with myocardial redox stress and hexose-6-phosphate-dehydrogenase enzymatic activity, suggesting cellular antioxidant mechanisms [17]. Enhanced oxidative stress has been reported to increase the ^18^F-FDG extraction fraction in myocardial metabolism [17]; thus, decreased cardiac ^18^F-FDG accumulation may be associated with cancer cachexia, which encourages the disruption of oxidative stress. Further studies are needed to investigate whether the relationship between cardiac ^18^F-FDG uptake and oxidative stress plays a crucial role in cancer cachexia.

In this study, visual assessment using a 5-point scale score was implemented to evaluate cardiac ^18^F-FDG uptake compared to SUV_max_. Our previous study also indicated the usefulness of visual assessment using PET to predict the outcome of immunotherapy for lung cancer [13]. We found that a lower cardiac ^18^F-FDG uptake on PET than that in normal hepatic lesions was associated with low nutrition, increased tumor activity, and cachexia, resulting in therapeutic resistance. Although there are only a few reports on visual assessment using a 5-point scale score for lung cancer, this measurement of glucose metabolism is not affected by different PET machines, and the assessment of ^18^F-FDG uptake is uniform among individual physicians. Although this five points score had been used for the assessment of ^18^F-FDG uptake in tumor lesions, little is known about the evaluation of cardiac ^18^F-FDG uptake. However, this scoring assessment is helpful for all lesions including non-cancerous areas, and clinical convenience is expected. Therefore, the criteria was applied to cardiac ^18^F-FDG uptake. Further study is warranted to elucidate the clinical convenience of visual assessment of ^18^F-FDG uptake by five points score.

In our study, one patient underwent SARS-CoV-2 vaccine, while, 14 patients did not receive its vaccine (data not shown). However, it remains unclear about the administration of SARS-CoV-2 vaccine in the remaining cases. As more than half of our patients were unknown about the receipt of SARS-CoV-2 vaccine, SARS-CoV-2 vaccine may affect the cardiac ^18^F-FDG accumulation, reading the increase of its uptake [18].

All patients in the current study had no history of any liver diseases. However, the relationship between cardiac ^18^F-FDG uptake and liver disease such as fatty liver has been described previously [19–21]. Some reported showed that non-alcoholic fatty disease is closely associated with reduced myocardial glucose uptake by ^18^F-FDG uptake [19–21]. Although ^18^F-FDG accumulation in liver an adipose tissue for cancer-associated cachexia has been investigated, the reduced ^18^F-FDG uptake in the liver and increased ^18^F-FDG uptake in visceral and subcutaneous fat were recognized in cancer patients with cachexia [22]. This report described that ^18^F-FDG uptake in liver and adipose tissue could be risk factor for identifying cancer-associated cachexia [22]. Moreover, it has been reported that the cardiac ^18^F-FDG uptake is dependent on cardiac energy milieu, such as plasma glucose, fatty acid, insulin, and ketone body [23, 24]. However, our ^18^F-FDG PET study was performed under 6 h fasting, thus, the energy milieu may be quite variable, causing variety of cardiac ^18^F-FDG uptake, independent of cachexia [23, 24].

Our study had several limitations. First, our study was a pilot investigation for a preliminary analysis, and our sample size was limited. This may have biased the results. However, our study included consecutive patients who received the first-line treatment for advanced NSCLC. Although all of our registered patients were suspected of having cachexia and were evaluated based on the definition of cachexia, approximately 40% of themwere identified as having definite cachexia. The consecutive selection of patients with and without cachexia was reasonable. Second, the first-line regimens were heterogeneous because they focused on the recruitment of patients with cachexia. Even if ICIs or EGFR-TKIs were initiated as first-line treatments, the 5-year OS rate was approximately 20% for both regimens [16, 17]. However, median PFS was longer in the EGFR-TKI group than in the ICIs group. The comparison of PFS in our study may have biased the survival analysis; however, the comparison of OS based on cardiac visual ^18^F-FDG uptake seemed certain. Finally, we did not investigate the effect of cancer cachexia on the biology of cardiac ^18^F-FDG accumulation. ^18^F-FDG accumulation within tumor cells has been described as closely correlated with the underlying biology by glucose metabolism, hypoxia and angiogenesis; thus, the increased ^18^F-FDG uptake was associated with poor outcome [1]. However, our data suggest that decreased uptake of cardiac ^18^F-FDG identified a progressive situation, such as cachexia, contrary to the phenomenon of ^18^F-FDG uptake within the primary tumor specimen. The biological mechanism of ^18^F-FDG uptake seemd to differ between cardiac and tumor tissues.

In conclusion, decreased visual cardiac ^18^F-FDG uptake identified poor nutritional status suggestive of cachexia in patients with advanced NSCLC. Therefore, decreased cardiac ^18^F-FDG uptake was identified as a potential factor for poor prognosis. Although the underlying mechanism of how cardiac ^18^F-FDG uptake is affected by cachexia remains unclear, the visual score for cardiac ^18^F-FDG accumulation is easily comprehensible, but not the not SUV_max_.

## Data Availability

No datasets were generated or analysed during the current study.

## Abbreviations

^18^F-FDG: Two-deoxy-2-[fluorine-18]-fluoro-d-glucose
PET: Positron emission tomography
NSCLC: Non-small cell lung cancer
SUV: Standardized uptake value
PD-1: Programmed death-1
EGFR-TKIs: Epidermal growth factor receptor-tyrosine kinase inhibitors
CT: Computed tomography
VOI: Volume of interest
SUV_max_: Maximum SUV
MTV: Metabolic tumor volume
TLG: Total lesion glycolysis
SUV_mean_: Average of 1.5 × SUV
5-PS: Five-point scale
CRP: C-reactive protein
NLR: Neutrophil count/lymphocyte count
PLR: Platelet count/lymphocyte count
PNI: Prognostic nutritional index
GPS: Glasgow prognostic score
PFS: Progression-free survival
OS: Overall survival
AC: Adenocarcinoma
ICIs: Immune checkpoint inhibitors
